# Predictive value of clinical and ^18^F-FDG-PET/CT derived imaging parameters in patients undergoing neoadjuvant chemoradiation for esophageal squamous cell carcinoma

**DOI:** 10.1038/s41598-022-11076-0

**Published:** 2022-05-03

**Authors:** Lisa Marr, Bernhard Haller, Thomas Pyka, Jan C. Peeken, Moritz Jesinghaus, Klemens Scheidhauer, Helmut Friess, Stephanie E. Combs, Stefan Münch

**Affiliations:** 1 Department of Radiation Oncology, Klinikum Rechts der Isar, Technical University of Munich (TUM), Ismaninger Str. 22, 81675 Munich, Germany; 2 Institute of Medical Informatics, Statistics and Epidemiology, Klinikum Rechts der Isar, Technical University of Munich (TUM), Ismaninger Str. 22, 81675 Munich, Germany; 3 Departement of Nuclear Medicine, Klinikum Rechts der Isar, Technical University of Munich (TUM), Ismaninger Str. 22, 81675 Munich, Germany; 4 Department of Nuclear Medicine, Inselspital Bern, Freiburgstr. 18, 3010 Bern, Switzerland; 5 German Cancer Consortium (DKTK), Partner Site Munich, Munich, Germany; 6 Institute of Radiation Medicine (IRM), Helmholtz Zentrum München (HMGU), Ingolstädter Landstraße 1, 85764 Oberschleißheim, Germany; 7 Institute of Pathology, Klinikum Rechts der Isar, Technical University of Munich (TUM), Ismaninger Str. 22, 81675 Munich, Germany; 8 Department of Surgery, Klinikum Rechts der Isar, Technical University of Munich (TUM), Ismaninger Str. 22, 81675 Munich, Germany

**Keywords:** Cancer, Oesophageal cancer

## Abstract

Aim of this study was to validate the prognostic impact of clinical parameters and baseline ^18^F-FDG-PET/CT derived textural features to predict histopathologic response and survival in patients with esophageal squamous cell carcinoma undergoing neoadjuvant chemoradiation (nCRT) and surgery. Between 2005 and 2014, 38 ESCC were treated with nCRT and surgery. For all patients, the ^18^F-FDG-PET-derived parameters *metabolic tumor volume* (*MTV*), *SUVmax, contrast* and *busyness* were calculated for the primary tumor using a SUV-threshold of 3. The parameter *uniformity* was calculated using contrast-enhanced computed tomography. Based on histopathological response to nCRT, patients were classified as good responders (< 10% residual tumor) (R) or non-responders (≥ 10% residual tumor) (NR). Regression analyses were used to analyse the association of clinical parameters and imaging parameters with treatment response and overall survival (OS). Good response to nCRT was seen in 27 patients (71.1%) and non-response was seen in 11 patients (28.9%). Grading was the only parameter predicting response to nCRT (Odds Ratio (OR) = 0.188, 95% CI: 0.040–0.883; *p* = 0.034). No association with histopathologic treatment response was seen for any of the evaluated imaging parameters including *SUVmax, MTV, busyness, contrast* and *uniformity*. Using multivariate Cox-regression analysis, the heterogeneity parameters *busyness* (Hazard Ratio (HR) = 1.424, 95% CI: 1.044–1.943; *p* = 0.026) and *contrast* (HR = 6.678, 95% CI: 1.969–22.643**;** *p* = 0.002) were independently associated with OS, while no independent association with OS was seen for *SUVmax* and *MTV*. In patients with ESCC undergoing nCRT and surgery, baseline ^18^F-FDG-PET/CT derived parameters could not predict histopathologic response to nCRT. However, the PET/CT derived features *busyness* and *contrast* were independently associated with OS and should be further investigated.

## Introduction

Esophageal cancer ranks sixth in cancer-related mortality and has caused more than 5,00,000 cancer deaths in 2018 worldwide^1^. Mortality rate of esophageal cancer is particularly high because most patients are diagnosed with locally advanced or metastatic disease^2^. Despite improvements in diagnostic and therapeutic techniques over the last decades the 5-year OS remains poor.

The standard treatment regimen for patients with locally advanced tumors suitable for surgery is neoadjuvant chemoradiation followed by surgery (nCRT + S)^3,4^. Thereby, nCRT can improve survival and R0 resection rate compared to surgery alone^5,6^. Patients who are unsuitable for surgery or decline surgery are generally treated with definitive chemoradiation (dCRT). Despite direct comparisons between dCRT and nCRT + S are often impaired by heterogeneous patient cohorts, it is known there is no significant difference for overall survival in patients with good response to (neoadjuvant) chemoradiation^7^. Therefore, pretherapeutic prediction of treatment response could guide the treating radiation oncologist to select personalized therapeutic strategies for each patient.

In patients with curative treatment intent, ^18^F-fluorodeoxyglucose positron emission tomography/computed tomography (^18^F-FDG-PET/CT) is typically used for staging and can help to detect distant metastases^8,9^. SUV-based parameters like maximum standardized uptake value (SUVmax) or metabolic tumor volume (MTV) have been analyzed for their ability to predict OS or histopathological response to chemoradiation in esophageal cancer patients^10–20^. These studies demonstrate contradictory results and interpretation of the data is also impaired by the fact, that most studies included different histologic subtypes. In recent years, studies focusing on heterogeneity parameters like *contrast* or *busyness* demonstrated that intratumor heterogeneity might be more suitable than SUV-based parameters in predicting treatment response or survival^11,21,22^. In addition, the contrast-enhanced CT-based parameter *uniformity* was associated with treatment response and survival in different types of cancer including esophageal carcinoma^23–26^.

In this study, the potential of the most promising ^18^F-FDG-PET/CT-based parameters to predict survival and histopathological response shall be further analyzed in a cohort of ESCC patients undergoing neoadjuvant chemoradiation and surgery.

## Patients and methods

### Baseline characteristics

Clinical data of 38 ESCC patients who underwent nCRT and esophagectomy with regional lymphadenectomy between 2005 and 2014, were retrospectively evaluated. 53% of the patients were male and median age was 62 years. The median craniocaudal tumor length was 5 cm (range 2–12 cm). 55% of patients had moderately differentiated tumors (G2) and 45% had poorly differentiated tumors (G3). Most patients (92%) had locally advanced tumor lesions (T3) and 87% of the patients had clinically suspected lymph node metastases (cN +). In addition, one patient had a supraclavicular lymph node metastasis that was classified as M1 (LYM) (Table1).

**Table 1:** Baseline characteristics.

Parameter	No. of patients (%) n = 38
**Sex**	
Male	20 (52.6)
Female	18 (47.4)
**Age (years)**	
Median	62
Range	33–75
**Tumor differentiation**	
G2	21 (55.3)
G3	17 (44.7)
**Tumor length (centimeter)**	
Median	5
Range	2–12
**T-stage**	
cT2	3 (7.9)
cT3	35 (92.1)
**N-stage**	
cN0	5 (13.2)
cN +	33 (86.8)
**Distant metastases**	
cM0	37 (97.4)
cM1	1 (2.5)

### Neoadjuvant chemoradiation

All patients received nCRT up with a total dose of 45 Gy (daily dose 1.8 Gy, 5 times/week) and concomitant platinum-based chemotherapy. 29 patients (76%) received chemotherapy with cisplatin and 5-fluorouracil (5-FU). In 8 patients the combination of oxaliplatin, 5-FU and cetuximab was used. One patient received combination chemotherapy with cisplatin and irinotecan. The median time interval between completion of neoadjuvant therapy and surgery was 39 days (range 9–84 days).

### ^18^F-FDG-PET

Prior to treatment a ^18^F-FDG-PET/CT was done either using the ´*Biograph 16*´ (Siemens Medical Solutions, Germany) or the *´SOMATOM Definition AS´* (Siemens Medical Solutions, Germany). In all patients the 3D-*Ordered Subsets Expectation Maximization* (OSEM3D) algorithm^27^, was used resulting in a comparable slice thickness of 5.3 mm and 4.1 mm.

Patients were instructed to fast for at least 6 h before injection of ^18^F-FDG (4.6 MBq/kg). Median time interval between injection of ^18^F-FDG and imaging was 69 min and imaging was done with 5–14 bed positions and an acquisition time of 1.5–4 min per bed position.

For calculation of PET-derived parameters (*SUVmax*, *MTV*, *busyness* and *contrast*) the region of interest (ROI) was semi-automatically generated in two consecutive steps: The ROI was automatically contoured using a SUV-threshold of 3. To assure, that only voxels containing tumor were included within the ROI all adjacent physiological ^18^F-FDG-avid structures beside the primary tumor area were then excluded manually. The resulting ROI included voxels with a SUV-uptake of ≥ 3 and assigned to the primary tumor. In the following the heterogeneity parameters were calculated automatically using Matlab software version 2018. The parameter *contrast*, which indicates differences between neighboring voxels, was calculated on 3D matrices as described by Tixier and colleagues^21^. In addition, the parameter *busyness*, which corresponds to the spatial frequency of intensity changes, was calculated as reported by Amadasun and King ^28^.

### Diagnostic contrast-enhanced computed tomography

Standardized pre-therapeutic contrast-enhanced (portal venous phase) CT-imaging with a maximum slice thickness of 5 mm was available in 30 patients (79%). The primary tumor in the esophagus was manually delineated using all available diagnostic information (endoscopy, endoscopic ultrasound (EUS) and ^18^F-FDG-PET scans). Voxels containing air or fluid within the esophagus were manually excluded. The image parameter *uniformity* quantifies the distribution of grey levels. Thus, *uniformity* is an indicator of image heterogeneity. Calculation of *uniformity* was done as described by Ganeshan and colleagues^29^ using IBEX (Imaging Biomarker Explorer) software^30^.

### Follow-up

All patients underwent regular follow-up examinations following international guidelines including physical examination, esophago-gastro-duodenoscopy and computed tomography.

### Histopathologic assessment

The basis for our analysis was the differentiation between patients with histopathological good response (R) and non-responders (NR). Therefore, resected specimen of all 38 patients were evaluated by extensive and standardized histomorphological workup as described by Becker et al.^31^. Complete tumor regression with 0% residual tumor was classified as grade 1a, subtotal tumor regression with < 10% residual tumor per tumor bed was classified as grade 1b, partial tumor regression with 10–50% residual tumor per tumor bed was classified as grade 2 and minimal or no tumor regression with > 50% residual tumor per tumor bed was classified as grade 3. For this analysis, good response (R) was defined as < 10% residual tumor (Becker grade 1a + 1b), while non-response (NR) was defined as ≥ 10% residual tumor (Becker grade 2 + 3).

### Statistics

Statistical tests were performed using the *SPSS Statistics softwar*e version 18.0.0 (IBM SPPS Statistics, Armonk, U.S.) and *R package* version 2019-07-25^32^. P-values < 0.05 were considered statistically significant. Univariate binary logistic regression analyses were conducted to evaluate the correlation of each parameter and response to therapy. OS was determined as the time between esophagectomy and last follow-up or death. Cox regression analyses were used to identify parameters predicting OS. The optimal cut-off value of significant parameters was determined by the maximally selected LogRank test^33^ and a test on association using the R package maxstat.test. Survival curves were estimated using the Kaplan Meier method.

### Ethics approval and consent to participate

The ethical committee of the Technical University of Munich has approved the retrospective study protocol (ethical vote N° 490/19 S). All patients gave their written informed consent for radiotherapy. All methods were performed in accordance with the relevant guidelines and regulations.

## Results

### Tumor response and imaging parameters

27 (71%) patients were classified as good responders (R) and eleven patients were classified as non-responders (NR) (29%). Initial median *MTV* was 14.6cm^3^ in responders, and 19.1cm^3^ in non-responders. Median *SUVmax* (^18^F-FDG) was 16.8 (R) and 17.6 (NR). Median *busyness*, *contrast* and *uniformity* were 1.53, 0.38, and 0.18 (R) and 2.82, 0.53 and 0.19 (NR), respectively. Using binary logistic regression analysis, neither *MTV* nor any other pre-therapeutic imaging parameter could significantly predict treatment response (Table 2).

**Table 2:** Binary logistic regression analysis for the ability of pre-treatment ^18^F-FDG-PET/CT parameters to predict response to nCRT in ESCC.

	Treatment response		Odds ratio (95% CI)	*p* value
	R	NR		
**Contrast**				
Median Range	0.38 (0.009–1.48)	0.53 (0–1.32)	0.612 (0.115–3.253)	0.564
**Busyness**				
Median Range	1.53 (0.22–4.97)	2.82 (0.61–5.85)	0.691 (0.425–1.122)	0.135
**Uniformity (× 1000)**				
Median Range	181.7 (93.8–269.1)	190.9 (154.1–220.1)	0.998 (0.977–1.020)	0.883
**SUVmax**				
Median Range	16.8 (3.5–36.8)	17.6 (6.4–39)	1.005 (0.916–1.102)	0.924
**MTV (cm**^**3**^**)**				
Median Range	14.6 (0.012–135.7)	19.1 (2.1–49.0)	1.005 (0.974–1.036)	0.763

*R* responders, *NR* non-responders.

### Tumor response and clinical parameters

Table 3 shows the association of clinical parameters and histopathological response to nCRT. No significant difference between responders and non-responders was seen for T-stage, N-stage, tumor length, age and sex. In contrast, tumor grading was significantly associated with treatment response. In summary, the odds of good tumor response was 5.3 times higher in patients with moderate tumor differentiation (G2) than in patients with poor tumor differentiation (G3) (OR = 0.188, *p* = 0.034).

**Table 3:** Binary logistic regression analysis for the ability of clinical parameters to predict response to nCRT in ESCC.

	Treatment response		Odds ratio (95% CI)	*p *value
	R	NR		
**Age (years)**				
Median Range	62 (47–75)	58 (33–67)	1.098 (0.996–1.211)	0.061
**Sex**				
Male	14 (70%)	6 (30%)	1.114 (0.273–4.548)	0.880
Female	13 (72%)	5 (28%)		
**Tumor length (cm)**				
Median Range	4.0 (2–10)	7.0 (2–12)	0.740 (0.535–1.022)	0.068
**Grading**				
G2	18 (86%)	3 (14%)	0.188 (0.040–0.883)	0.034
G3	9 (53%)	8 (47%)		
**N-stage**				
cN0	4 (80%)	1 (20%)	0.575 (0.057–5.814)	0.639
cN +	23 (70%)	10 (30%)		
**T-stage**				
cT2	3 (100%)	0 (0%)	0.000 (0.000-NA)	0.999
cT3	24 (69%)	11 (31%)		

*R* responders, *NR* non-responders.

### Survival prediction

With a median follow-up of 68 months for surviving patients, median OS for all patients was 40 months. The 3-year cumulative survival was 56% and 5-year survival was 46%. Results concerning the prognostic value of all analyzed parameters are shown in Table 4.

**Table 4:** Results of Cox-regression analysis for OS in ESCC treated with nCRT and esophagectomy.

Parameter	Hazard ratio (95% CI)	*p *value
Busyness	1.564 (1.196–2.044)	0.001
Contrast	9.340 (2.987–29.207)	< 0.001
MTV (cm^3^)	1.000 (0.983–1.016)	0.958
SUVmax	0.934 (0.881–0.989)	0.019
Uniformity (× 1000)	1.005 (0.993–1.016)	0.439
Age	1.004 (0.957–1.054)	0.861
Sex (female vs. male)	0.647 (0.282–1.487)	0.305
Grading (G3 vs. G2)	1.968 (0.841–4.606)	0.118
N-stage (N1 vs. N0)	0.943 (0.277–3.210)	0.925
T-stage (T3 vs. T2)	0.634 (0.187–2.149)	0.464
Tumor length	0.991 (0.816–1.204)	0.930

Using a univariate Cox-regression model, none of the clinical parameters (age, sex, grading, T-stage, N-stage and tumor length) showed good predictive performance for OS, whereas the textural features *busyness* (Hazard Ratio (HR) = 1.564, *p* = 0.001), *contrast* (HR = 9.340, *p* < 0.001) and *SUVmax* (HR = 0.934, *p* = 0.019) were significantly associated with OS. While each increasement for the parameters *busyness* and *contrast* by “1” (*busyness*) and “0.1” (*contrast*) was associated with a 1.564 times (*busyness*) and a 1.250 times (*contrast*) higher risk of death, each increasement by “1” for the *SUVmax* was associated with a 1.07 times lower risk of death. Because of a significant high correlation (Pearson´s index = 0.899, *p* < 0.001) between *busyness* and *contrast*, both parameters were not tested within the same multivariate cox-regression analysis. When using multivariate cox-regression models including the parameters *SUVmax* and *busyness* or *SUVmax* and *contrast*, the impact of *SUVmax* was not statistically significant anymore (*p* = 0.301/*p* = 0.224) while the parameters *busyness* (HR = 1.424, 95% CI: 1.044–1.943; *p* = 0.026) and *contrast* (HR = 6.678, 95% CI: 1.969–22.643; *p* = 0.002) were independently associated with OS. Thereby, an increasement by “1” (*busyness*) or “0.1” (*contrast*) was still associated with a 1.424 times (*busyness*) and a 1.209 times (*contrast*) higher risk of death.

**Figure 1:**
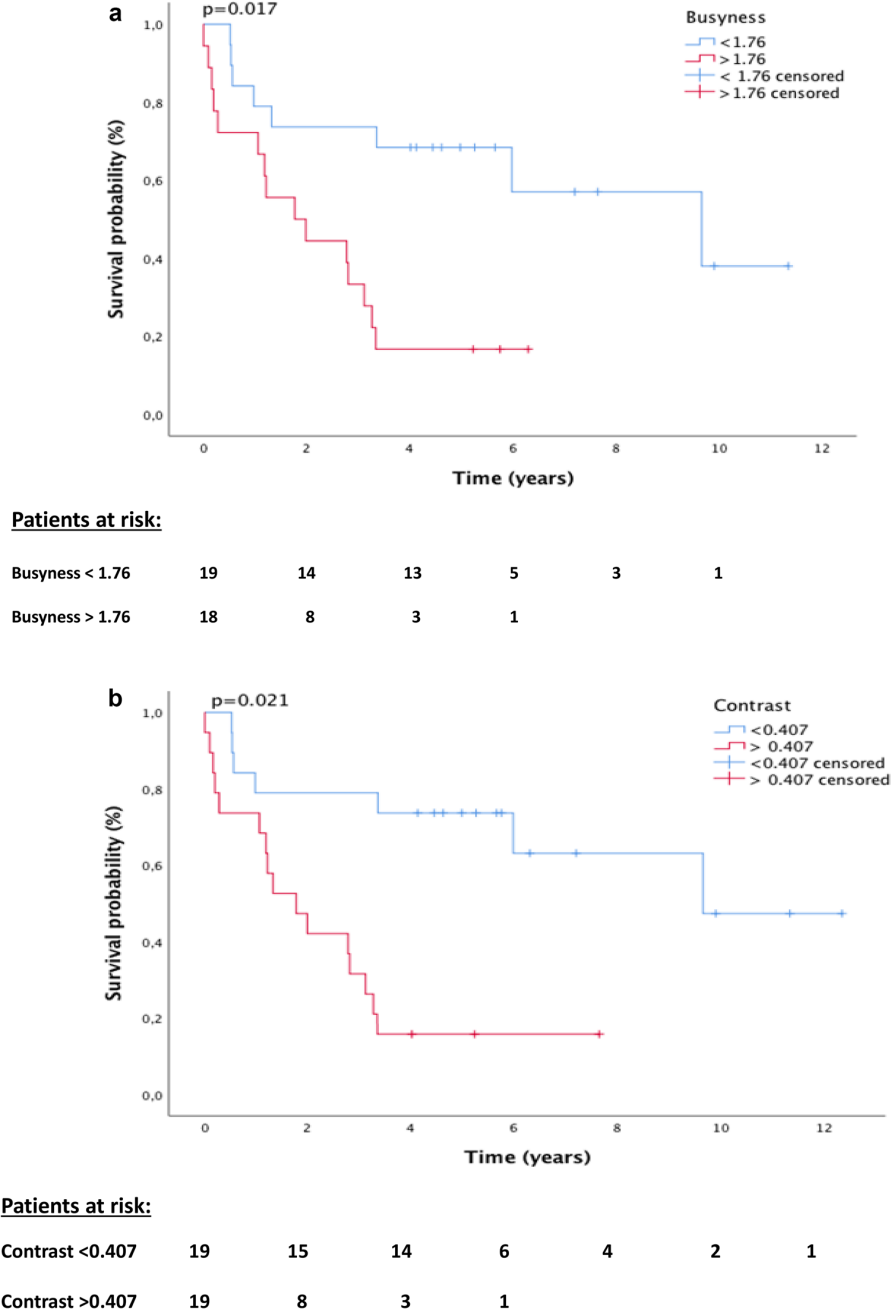
(**a**) Kaplan–Meier survival curves of the OS stratified by the textural parameter *busyness*. (**b**) Kaplan–Meier survival curves of the OS stratified by the textural parameter *contrast*.

Using the maximally selected LogRank test optimal cut-off values of 0.407 (*contrast*) and 1.76 (*busyness*) were calculated and used for further analysis. Median OS was 1.8 years in the subgroup of patients with tumor *contrast* > 0.407 and 9.7 years for patients with tumor *contrast* < 0.407 (*p* = 0.021) (Fig. 1a). Similarly, *busyness* of < 1.76 was associated with a better outcome and a median survival of 9.7 years than *busyness* of > 1.76 (1.8 years; *p* = 0.017) (Fig. 1b). Finally, there was no statistically significant correlation between OS and the ^18^F-FDG-PET/CT based image parameters *MTV* and *uniformity*.

## Discussion

Assessment of prognosis and response to therapy prior to treatment plays an increasing role in patient management as well as treatment planning. Being able to predict prognosis and response to nCRT based on a standard pre-treatment imaging would be practicable, cost- and timesaving in clinical routine. In this context, the value of baseline PET-based parameters for prediction of treatment response and prognosis has been promisingly described in the literature for different tumor entities^34–38^. However, regarding ESCC, only few data showing conflicting results are available. Therefore, we validated the impact of ^18^F-FDG-PET/CT derived textural features for prediction of treatment response to neoadjuvant chemoradiation in a cohort of only ESCC patients treated at our institution. Neoadjuvant treatment was very homogenous with all patients receiving neoadjuvant radiotherapy with a total dose of 45 Gy and simultaneous platin-based chemotherapy. This is an important aspect because the efficiency of chemoradiation differs between the two histological subtypes^6^ and radiation doses can also affect patients´ response to chemoradiation.

None of the analyzed pretreatment ^18^F-FDG-PET/CT parameters including *SUVmax*, *MTV*, *contrast*, *busyness* and *uniformity* were associated with histopathological response to nCRT. Regarding histopathological parameters, patients with moderate differentiated tumors showed higher response rates than patients with poorly differentiated tumors. However, in terms of OS, a lower *SUVmax*, higher *contrast* and higher *busyness* were associated with inferior OS.

The PET derived SUV quantifies the FDG accumulation rate and is particularly high in tissues with pathological processes such as inflammation or tumor tissue. However, the typically used image parameter *SUVmax* does not represent the glucose metabolism of the entire tumor but indicates the highest value within a defined region of interest. While pretreatment *SUVmax* was associated with response to chemoradiation in two studies primarily including patients with adenocarcinoma^17,18^, results for patients with squamous cell carcinoma are heterogenous^10,16,20,21^. In a study by Zhang and colleagues^10^ treatment response was assessed 4 weeks after chemoradiation in 48 ESCC patients. While the reduction of *SUVmax* between baseline PET and an interim-PET after 40 Gy was predictive for treatment response, no significant association was seen for baseline *SUVmax* alone. In addition, another study mainly including ESCC patients (> 70%) also found no association between pretreatment PET and response to chemoradiation^16^. While this is in line with our results, in two other studies with mainly ESCC patients, lower baseline *SUVmax* was associated with higher rates of clinical complete response^20,21^. In contrast to the presented data, in all these studies, treatment response was assessed by computed tomography using RECIST (Response Evaluation Criteria in Solid Tumours)^39^ and not by histopathological workup.

There was no independent association between *SUVmax* and OS. This is in line with several other studies analyzing the predictive value of baseline *SUVmax* in patients with predominantly ESCC^12,13,40,41^. When looking to other survival endpoints like relapse-free survival or disease-free survival, contradictory results are presented in the literature^12,42^. While patients with a *SUVmax* > 7 of the primary tumor tended to have a lower RFS after R0-esophagectomy than patients with a *SUVmax* < 7 in a study by Shimizu and colleagues^42^, in another study by Lemarignier et al.^12^ a higher baseline *SUVmax* of the primary tumor predicted longer disease-free survival in ESCC patients after chemoradiotherapy. Beside the mix-up of different histologic subtypes, contradictory results seen in the literature, might be partly explained by the fact that *SUVmax* generally depends on the assessment method and the technical standard of the PET equipment ^43,44^.

As shown *MTV* was not found to predict treatment response or OS in ESCC patients undergoing neoadjuvant chemoradiation. In contrast to our results large *MTV* was associated with shorter OS in previous trials^12,13,41^. However, while we used an absolute SUV threshold of 3 to determine *MTV*, all these studies used just relative or even individual SUV thresholds. Conforming with our data, in a study by Nakajo and colleagues^11^, who used an absolute SUV threshold of 2.5, *MTV* was also not associated with OS in a multivariate analysis. Thereby, different *MTV* definitions may impair the comparability of results and individual SUV threshold seem to be more suitable than absolute thresholds. In addition, most patients included in the mentioned studies were treated with either definite chemoradiation or esophagectomy, while all patients in our study underwent neoadjuvant chemoradiation and subsequent surgery. It remains unclear, to what extent the prognostic significance of *MTV* might be affected by treatment regimen. Regarding response to chemoradiation, our study confirms the result of an earlier study, in which also no association was seen between *MTV* and treatment response^10^.

Although *SUVmax* and *MTV* are still the most commonly used PET derived image parameters, recent studies increasingly focus on characterization of local tumor heterogeneity. Generally, heterogeneity within the tumour microenvironment is caused by hypoxia, necrosis and cellular proliferation. These factors are associated with adverse tumour biology and are considered as hallmarks of malignancy. Thus, textural parameters that correspond to increased heterogeneity (e.g. decreased *uniformity*) have been associated with poorer overall survival. Therefore, the prognostic impact of the three heterogeneity parameters *busyness*, *contrast* and *uniformity* was further evaluated in this study. None of the heterogeneity parameters was predictive for treatment response, which is in line with the results described by Tixier and colleagues^21^. They analyzed the impact of different textural features to predict response to chemoradiation in patients with mostly ESCC and no significant association was seen between treatment response and the parameters *contrast* and *busyness*.

In contrast to a study by Ganeshan and colleagues^24^, no association was seen between OS and uniformity in our study. However, comparison of the results is strongly impaired by the fact, that the authors included patients with mostly adenocarcinoma and used non-contrast-enhanced CT data for imaging analysis. While there are very limited data regarding the prognostic impact of heterogeneity parameters in patients with ESCC, association of baseline busyness or contrast and survival has already been described in several other tumor entities including rectal cancer, non-small cell lung cancer and hypopharyngeal carcinoma^35,37,45^.

Because parts of the analysis were done retrospectively, this study has some limitations. First, the study was conducted at a single institution and the total number of patients is limited. This clearly compromises any generalization of results and conclusions. However, the limited number of patients may be more than outweighed by the fact, that only patients with ESCC were included. Secondly, PET acquisition and reconstruction have been developed since 2005, which may further limit the comparability of imaging parameters. But even if the repeatability and reproducibility of the heterogeneity measurement depends on the image processing method and the reconstruction algorithm, local heterogeneity parameters are generally regarded as particularly robust^46^. Third, the imaging parameters in our study were defined using a SUV threshold of 3. This limits the comparability to other studies, where different SUV thresholds were used. However, the optimum threshold depends amongst others on the clinical setting, tumor characteristics and on the type of PET machine and therefore no standard threshold has been established for universal clinical use so far.

Although the predictive potential of different ^18^F-FDG-PET/CT derived textural features has successfully been shown for several types of malignancies, this study confirms the current limitation of baseline PET-CT guided response prediction in ESCC patients. This even applies to a particularly homogeneous patient population with only one histological subtype and homogeneous radiotherapy regime.

In conclusion, our data do not support the use of PET/CT derived imaging parameters to predict response to nCRT in patients with ESCC. However, the study supports the thesis that textural analysis of local heterogeneity in the tumor´s tracer uptake might also be suitable to predict survival and should be further evaluated.

## Data Availability

The datasets used and/or analysed during the current study available from the corresponding author on reasonable request.
